# Nutrient Status and Intakes of Adults with Phenylketonuria

**DOI:** 10.3390/nu16162724

**Published:** 2024-08-15

**Authors:** Eva Venegas, Simone Langeveld, Kirsten Ahring, Rosa Benitez, An Desloovere, Elena Dios, Eva Gómez, Alvaro Hermida, Cyril Marsaux, Patrick Verloo, Maria-Luz Couce

**Affiliations:** 1 Endocrinology and Nutrition Department, Hospital Universitario Virgen del Rocío, 41013 Sevilla, Spain; evam.venegas.sspa@juntadeandalucia.es (E.V.); r.beniteza@hotmail.com (R.B.); mariae.dios.sspa@juntadeandalucia.es (E.D.); 2 Danone Research & Innovation, 3584 CT Utrecht, The Netherlands; simone.langeveld@danone.com; 3 Department of Phenylketonuria, Copenhagen University Hospital, 2100 Copenhagen, Denmark; kirsten.ahring@regionh.dk; 4 Department of Pediatric Neurology, Center for Inherited Metabolic Disorders, University Hospital Ghent, European Reference Network for Hereditary Metabolic Disorders (MetabERN), 9000 Ghent, Belgium; an.desloovere@uzgent.be (A.D.); patrick.verloo@ugent.be (P.V.); 5 Unit for Diagnosis and Treatment of Congenital Metabolic Disorders, University Clinical Hospital of Santiago de Compostela, Health Research Institute of Santiago de Compostela (IDIS), European Reference Network for Hereditary Metabolic Disorders (MetabERN), 15706 Santiago de Compostela, Spain; dieteva@hotmail.es (E.G.); alvaro.hermida.ameijeiras@sergas.es (A.H.); maria.luz.couce.pico@sergas.es (M.-L.C.)

**Keywords:** phenylalanine hydroxylase deficiency, nutritional status, amino acid, micronutrient, cognitive wellbeing

## Abstract

A phenylalanine-restricted diet, supplemented with protein substitutes (PSs), remains the cornerstone of phenylketonuria (PKU) management. However, adherence is challenging in adulthood, and data on the nutritional status of early and continuously treated adults with PKU (ETAwPKU) are scarce. A total of 34 ETAwPKU (16 females; mean ± SD, age: 28 ± 9 years, phenylalanine concentration: 847 ± 285 µmol/L) and 34 age- and sex-matched control subjects were compared regarding their blood nutrient status, self-reported dietary intake, and cognitive wellbeing. Though diet adherence varied, all ETAwPKU were taking a PS. No significant differences were found for blood DHA, calcium, ferritin, transferrin, and zinc concentrations. However, selenium and ubiquinone concentrations were 16% and 29% lower in ETAwPKU, respectively (*p* < 0.01 and <0.0001). Vitamin concentrations (D, B12, B6, and folic acid) were significantly higher in ETAwPKU except for alpha-tocopherol. Amino acid (AA) concentrations differed between ETAwPKU and controls: they were significantly lower for 12 AAs and higher for phenylalanine and glycine. ETAwPKU had a significantly higher intake of most minerals and vitamins, except for niacin and phosphorus (no difference). Depending on the nutrient, PSs represented 52–100% of patients’ daily intake and 19% of total daily energy intake. Compared with controls, ETAwPKU scored significantly lower in three of the four subscales of the cognitive wellbeing questionnaire. Overall, the blood DHA and micronutrient status of ETAwPKU was adequate, except for selenium, with higher intakes than controls for most micronutrients. Patients relied heavily on PSs to meet the recommended intakes for protein, DHA, and micronutrients. The potential clinical impact of differences found in AA status should be further studied.

## 1. Introduction

Phenylketonuria (PKU) is a rare inherited metabolic disorder, where phenylalanine (Phe) is not appropriately converted into tyrosine, due to a deficiency of the enzyme Phe hydroxylase. As a result, Phe accumulates in the blood and other tissues. If PKU is left untreated, the excessive Phe concentrations in the brain can cause devastating neurocognitive deficits [1,2]. This can be successfully prevented by early treatment, which has been made possible in many parts of the world thanks to newborn screening [3,4,5].

PKU treatment aims to maintain Phe concentrations in the therapeutic target range (i.e., <360 µmol/L for life according to US guidelines [5] or <360 µmol/L for children < 12 years and <600 µmol/L thereafter according to European guidelines [4]). For the majority of patients, such metabolic control is achieved through a strict, lifelong, low-protein diet, which prevents excessive Phe intake. This restrictive diet needs supplementation with a Phe-free or low-Phe protein substitute, i.e., a protein replacement formula, which usually contains additional tyrosine, micronutrients, essential fatty acids, and long-chain polyunsaturated fatty acids, to ensure that infants and children with PKU can meet their nutrient requirements for growth and development [6]. In adulthood, protein substitutes continue to play a key role in maintaining a healthy nutritional status.

Although successful, dietary management of PKU represents a heavy burden for patients as well as their families, and it is well known that diet adherence deteriorates in teenagers and adults, leading to Phe concentrations commonly above 600 µmol/L [7,8]. Non-adherence to protein substitute prescriptions further puts patients at risk of nutritional inadequacies [9,10]. Additional factors can affect the nutrient status of patients with PKU, such as the bioavailability of different nutrient sources, differences in compositions of protein substitutes available to patients, different management approaches between metabolic centers, or even access to a multidisciplinary team [4].

Surprisingly little has been published on the nutrient status of adults with PKU. A relatively recent systematic review and meta-analysis [11] found significantly lower levels of docosahexaenoic acid (DHA), eicosapentaenoic acid (EPA), and cholesterol in patients with PKU compared to healthy controls [11]. No significant differences were observed for vitamins B12, E, and D or zinc, calcium, iron, and magnesium. However, children and adults could not be analyzed separately, and some studies were quite old (<2001), i.e., likely did not reflect later improvements in protein substitute compositions. Furthermore, interpretation for some nutrients (e.g., selenium) was challenging due to the considerable heterogeneity between studies, and for others (e.g., vitamin B6, uridine), data were lacking [11]. When comparing adults with PKU who continued to take their protein substitutes regularly with irregular users, Hochuli et al. [9] found that non-adherent adults had lower intakes of protein and certain micronutrients, some falling below recommended intakes, as well as lower blood vitamin B12 and tyrosine levels, and trends toward lower concentrations of branched-chain amino acids. Reported values were typically in the range considered normal by the hospital’s laboratory (albeit at the lower end). This study lacked a control group (i.e., adults without PKU).

Additional quantitative insights regarding the nutrient status and intakes of adults with PKU are needed to improve dietary management. Therefore, the main objective of this study was to compare blood micro- and macronutrient concentrations of early and continuously treated adults with PKU, taking at least one dose of protein substitute per day, with age- and sex-matched control subjects without PKU. Furthermore, we compared the nutritional intakes as well as measures of cognitive wellbeing between both groups.

## 2. Materials and Methods

### 2.1. Study Design

This was a prospective, multicenter, case-control, cross-sectional study conducted at four metabolic centers (Sevilla, Santiago de Compostela, Ghent, and Copenhagen) and one research facility (Utrecht) (ClinicalTrials.gov Identifier: NCT03858101). Enrolled subjects attended a single study visit for the collection of a blood sample. In addition, they were asked to complete a 3-day food diary and additional questionnaires at home (see Section 2.3: Outcome Measures).

The study was carried out in accordance with the ‘World Medical Association Declaration of Helsinki’ (64th WMA General Assembly, Fortaleza, Brazil, October 2013), the International Conference on Harmonization (ICH) guidelines for Good Clinical Practice (GCP, November 2016), as appropriate for nutritional products, and the local legislation of the country in which the research was conducted. All study documents were reviewed and approved by the applicable ethics committees.

### 2.2. Study Population

The planned enrolment for this study was 40 adults with PKU using a protein substitute and 40 age- (±3 years), and sex-matched control subjects without PKU. Participants with PKU had to be diagnosed by newborn screening and started on a low-Phe diet before one month of age. In addition, patients were only eligible if they had been using at least one protein substitute daily for the last 6 months. Patients using BH4 or other drugs that could interfere with the main outcome were excluded. Control subjects were not eligible if following a special diet (e.g., vegan diet). Other than vitamin D, which is commonly prescribed in both patients and the general population, patients were not allowed to take additional multivitamin/mineral supplements during the study. Finally, only one member per household was allowed to participate. Informed consent was obtained from all patients included in the study.

### 2.3. Outcome Measures

#### 2.3.1. Main Outcome: Blood Nutrient Status

The main outcome was the blood nutrient status, as reflected by blood concentrations of multiple nutrients and biochemical parameters. Venous blood samples were obtained from all participants in a fasting state during the study visit. The laboratory testing included selected vitamins (pyridoxal 5′-phosphate [PLP, the active form of vitamin B6], B12, folic acid, 25-OH vitamin D, and alpha-tocopherol [vitamin E]), minerals (calcium, ferritin, transferrin, magnesium, selenium, and zinc), lipids (DHA and EPA, from EDTA plasma and red blood cells), as well as a complete amino acid profile and other biochemical parameters (creatinine, homocysteine, uridine, and ubiquinone). Blood samples were analyzed by Reinier Haga Medisch Diagnostisch Centrum (Delft, the Netherlands) and the analytical science lab of Danone Research & Innovation (Utrecht, The Netherlands) (see also Methods S1).

#### 2.3.2. Other Outcomes

Other outcomes were nutrient intake and cognitive wellbeing, assessed through a self-reported diary and questionnaire during the week following the study visit.

The nutrient intake was assessed using a 3-day food diary recorded by the subjects themselves on three consecutive days (two weekdays and one weekend day). From this diary, the nutrient intake was calculated by each study site using dietetic software tools (Odimet version 2022 in Spain; Dankost Pro version 2.4.14.12596 in Denmark, and Evry version 6.7.7.0 in Belgium and The Netherlands). Most dietetic software could not provide DHA and EPA from natural foods, and thus, the total intake data of these two nutrients are missing for the majority of subjects. Furthermore, as most dietetic software could not provide amino acid intakes from natural foods, this was determined for Phe only, by hand, as follows: for fruits, Phe content = 3% of protein content; for vegetables (fresh/frozen, no flour coating), Phe content = 4% of protein content; for all other foods (including dairy products, flour, pulses, meat, or fish), Phe content = 5% of protein content [12]. Subjects were instructed not to change dietary habits between the study visit and the recording of the diet diary. Nutrient intake levels were categorized as low, adequate, or high, according to the daily recommended intake (DRI) values and tolerable upper intake levels derived by the European Food Safety Authority (EFSA) (Table S1). For the PKU group only, nutrient intakes from protein substitutes were also expressed as a percentage of the total dietary intake.

Subjective cognitive wellbeing was evaluated using the Functional Assessment of Cancer Therapy (FACT)-Cognitive Function (FACT-Cog) questionnaire (Version 3). The FACT-Cog questionnaire contains 37 items with 4 subscales: perceived cognitive impairments (score range 0 to 72), perceived cognitive abilities (score range 0 to 28), comments from others (score range 0 to 16), and impact on quality of life (score range 0 to 16) [13]. For all subscales, a higher score translates into a better quality of life.

### 2.4. Statistical Analyses

Statistical analyses were performed using SAS^®^ Version 9.4_TS1M3 or higher in SAS Life Science Analytics Framework Version 5.3 or higher for LIN X64, SAS Institute Inc., Cary, NC, USA. Statistical analyses were performed on the per-protocol population, defined as all subjects without major protocol violations who had a viable blood sample in the fasting state at Visit 1. Due to the exploratory nature of the study, no adjustments were made for multiplicity in any of the analyses. A 2-sided *p*-value of <0.05 was considered statistically significant. A descriptive analysis was completed for all study outcomes.

#### 2.4.1. Main Outcome

For the main outcome parameter, a paired *t*-test or a Wilcoxon Signed-Rank test was used to test for differences in nutrient/biochemical parameter concentration between the PKU and matched non-PKU groups. A subgroup analysis was completed using an independent *t*-test to test for differences in the blood nutrient concentration between PKU subjects with good metabolic control (defined as blood phenylalanine concentration ≤ 600 µmol/L) and poor metabolic control (blood phenylalanine concentration > 600 µmol/L).

#### 2.4.2. Other Outcomes

Differences in the nutrient intake between the PKU and non-PKU groups were tested using the Wilcoxon Signed-Rank test. In addition, the frequency and percentage of subjects with low, adequate, and high intake levels were presented per group (PKU and non-PKU) without further statistical testing.

Differences in scores on the FACT-Cog subscales between the PKU and non-PKU groups were tested using a paired *t*-test (‘perceived cognitive abilities’ and ‘quality of life’ subscales) or Wilcoxon Signed-Rank test (‘perceived cognitive impairments’ and ‘comments from others’ subscales). Additionally, linear regression analysis was performed as the exploratory outcome on the perceived cognitive impairments and perceived cognitive abilities subscales, with (1) the Phe concentration, (2) the average Phe level from 2 years ago, or (3) the Phe/tyrosine ratio as a predictor. These three analyses were completed in three ways: (1) without confounders, (2) with the study site as the confounder, and (3) with the study site plus age as confounders.

#### 2.4.3. Sensitivity Analysis

The main analysis was repeated, excluding adults with PKU who took at least one glycomacropeptide [GMP]-based protein substitute (N = 7) or large neutral amino acid [LNAA] supplement (N = 2) daily and their matched controls. An additional sensitivity analysis repeated the main analysis excluding subjects with a BMI of ≥30 kg/m^2^ (PKU, N = 5; non-PKU, N = 2) and matched PKU or non-PKU subjects.

## 3. Results

A total of 36 patients with PKU and 35 matched control subjects were recruited into the study between April 2019 and April 2022. One patient who had not fasted was excluded from the study. Furthermore, one other patient was excluded from the per-protocol analyses due to the usage of branched-chain amino acid and multivitamin supplements, which violated the study protocol. The baseline characteristics of the analyzed cohorts are provided in Table 1. In short, the PKU and non-PKU groups were well-matched in terms of age, sex, and BMI. All but one patient had classical PKU. Patients’ average blood Phe concentrations from the previous year were highly variable (median: 648.9 µmol/L, range 241.0–1479.0 µmol/L), while historical compliance with the low-Phe diet was typical of an adult clinic setting (e.g., 26% somewhat compliant and 70% frequently/always compliant according to their metabolic team). At the time of the study, two-thirds of the patients were considered compliant with the Phe-restricted diet.

### 3.1. Blood Concentrations of Nutrients and Other Biochemical Parameters

The blood alpha-tocopherol (vitamin E) concentration was significantly lower, whereas the folic acid, 25-OH vitamin D, vitamin B12, and PLP (vitamin B6) concentrations were all significantly higher in adults with PKU compared with their matched controls (Table 2). The selenium concentration was significantly lower in the PKU group compared with controls (relative difference: −16%). While the difference in magnesium concentrations between groups was statistically significant, it was very small (mean [SD] difference PKU vs. controls = +0.02 [0.06] mmol/L or +2% difference). No statistically significant differences were found for calcium, ferritin, transferrin, or zinc (Table 2). The blood concentrations of ubiquinone and uridine were significantly lower in the PKU group compared with the control group (Table 2). Results from the sensitivity analyses were similar, except that the difference for magnesium was no longer statistically significant.

No significant differences were found for blood DHA or EPA between the PKU and matched control subjects (Table 2). However, the sensitivity analyses revealed significantly lower plasma EPA concentrations (mg/L) and RBC EPA (%) in the PKU group compared with the control group. This is likely explained by one subject with PKU, both obese and taking LNAAs (and, therefore, excluded in the sensitivity analyses) who had very high EPA blood concentrations.

Blood concentrations of creatinine and homocysteine were significantly lower in the PKU group compared with the control group (Table 2). Results from the sensitivity analyses were similar.

Subjects with PKU had significantly lower concentrations of 12 amino acids compared with subjects without PKU (including eight of nine non-Phe large neutral amino acids [LNAAs] such as branched-chain amino acids, Table 3). As expected, the Phe concentration was much higher in subjects with PKU; however, glycine was also significantly higher compared with subjects without PKU. No other significant differences were observed for the remaining five amino acids. Also as expected, the Phe/tyrosine ratio was significantly higher in the PKU group than in the non-PKU group (mean [SD]: 21.87 [9.81] vs. 0.98 [0.18], *p*-value < 0.0001). Results from sensitivity analyses were largely similar when excluding subjects with BMI ≥30 kg/m^2^, except that the difference was no longer statistically significant for asparagine, or when excluding subjects who took GMP protein substitutes or LNAA supplements (the difference was no longer statistically significant for asparagine, proline, and valine). This might be due to the smaller sample sizes and consequent decrease in statistical power.

Patients’ metabolic control was highly variable (mean ± SD Phe concentration: 847 ± 285 µmol/L, range: 223–1408 µmol/L). A subgroup analysis was performed comparing patients with good metabolic control (Phe concentration ≤ 600 µmol/L; N = 6) and poor metabolic control (Phe concentration > 600 µmol/L; N = 28). Results of the independent *t*-test showed no statistically significant differences between the two subgroups for any of the nutrient or biochemical parameters measured, with the obvious exception of the Phe concentration and the Phe/tyrosine ratio, both *p*-values < 0.0001.

### 3.2. Nutritional Intake

The daily Phe intake was highly variable in the PKU group (range: 3 to 29 mg per kg body weight per day; median: 14). As expected, the Phe intake was significantly lower in the PKU group (*p*-value < 0.0001; N = 29 pairs).

The vitamin intake was significantly higher in the PKU group, apart from niacin (no statistically significant difference) (Table 4). Most subjects with PKU (79–94%) met EFSA’s DRI for vitamins B2, B5, B6, C, and E, though 37% of subjects with PKU had intakes below the DRI for vitamin D and B12. In the non-PKU group, none of the subjects met the DRI for vitamin D, and fewer individuals (25–59%) met the DRIs for the other vitamins compared with the PKU group.

The mineral intake was also significantly higher in the PKU group, with the exception of phosphorus (no statistically significant difference) (Table 4). Most subjects with PKU (60–100%) met EFSA’s DRI for the minerals assessed, apart from magnesium, where 20% of subjects had low intakes and 66.7% had high intakes. In addition, 27% of subjects with PKU had zinc intakes above EFSA’s tolerable upper intake level. In the non-PKU group, fewer subjects (12–41%) met the DRIs for minerals, except for phosphorus (97% met the DRI).

The EPA intake was significantly lower in the PKU group, while no statistical differences were found for the other nutrients (DHA, energy, fiber, and total protein; Table 4).

For patients with PKU, protein substitutes represented the main source of protein (66%; N = 30; Table 5), vitamins (>60% for B vitamins, up to 84% for B12, and almost 90% for vitamin D; N = 30), minerals (58% for copper and up to 74% for zinc; N = 30), and DHA (100%; N = 6) and EPA (75%; N = 4), while they contributed to about 20% of the total daily energy intake. Fiber intake mainly came from natural foods.

### 3.3. Subjective Cognitive Wellbeing

The PKU group had significantly lower scores than the non-PKU group for the subscales ‘quality of life’, ‘perceived cognitive abilities’, and ‘comments from others’, while no statistically significant difference was found for the ‘perceived cognitive impairments’ subscale (Table 6). Using a linear regression analysis, no statistically significant correlation was found between the perceived cognitive impairment subscale and the current Phe levels, the average Phe levels from 2 years ago, or the current Phe/tyrosine ratio. However, the perceived cognitive abilities score was statistically significantly and inversely correlated with the Phe/tyrosine ratio (Figure S1), and this inverse relationship remained statistically significant after correcting both for the study site and age (beta estimate –0.27, *p*-value = 0.0187).

## 4. Discussion

This study evaluated the nutrient status and intakes of adults with PKU, who have been on diet therapy since infancy. Our patient sample was typical of an adult PKU clinic. The metabolic control was highly variable, as was the daily Phe intake, which is not surprising for a cohort of adult patients with varying baseline Phe tolerances, lifestyles, and degrees of adherence to dietary prescriptions. We found that, compared with controls, adults with PKU had higher blood concentrations of folic acid and vitamins D, B6, and B12, whereas they had lower concentrations of vitamin E, selenium, ubiquinone, EPA, several amino acids, uridine, as well as creatinine, and homocysteine. Though statistically significant, the difference in magnesium concentration was minimal and likely not clinically relevant. Furthermore, no significant differences were found between groups for blood DHA, calcium, ferritin, transferrin, and zinc concentrations. Despite the Phe-restricted diet, nutrient intakes were adequate for the majority of patients, with most of the protein, DHA, and micronutrient intakes provided by protein substitutes. Of note, substantially fewer controls were able to meet EFSA’s DRIs for micronutrients.

To date, only a handful of studies with a control group have focused specifically on adult patients on diet monotherapy. Lage et al. [14] found significantly lower plasma DHA and EPA concentrations in a group of 22 adults with PKU, compared with matched controls; however, the protein substitutes used by patients did not contain any long-chain polyunsaturated fatty acids (LCPUFA). DHA and EPA are key structural components of neuronal membranes, which, in addition, contribute to the maintenance of normal blood pressure, normal blood triglyceride levels, normal cardiac function, as well as normal vision [15,16]. However, the main food source for these LCPUFAs is oily fish, which is excluded in the Phe-restricted diet. Although this might be compensated by the consumption of vegetable fats rich in precursor omega-3 fatty acids, such as α-linolenic acid (ALA), endogenous synthesis of DHA and EPA from ALA is limited [17,18,19]. Furthermore, in PKU, elevated concentrations of by-products of Phe metabolism are suggested to further inhibit DHA synthesis [20]. Multiple studies have highlighted a poorer LCPUFA status in patients across various age groups [21], and thus, protein substitutes are now commonly fortified with DHA and sometimes EPA. This could explain the lack of the difference in erythrocyte omega-3 fatty acid concentrations (including DHA and EPA) reported by Htun et al. [22] between their cohort of 43 early-treated adults with PKU and controls. In our study, most protein substitutes contained DHA, and they contributed to 100% of the total intake for patients. In contrast, almost none of the protein substitutes used were fortified with EPA, which could be why we observed lower EPA concentrations both in plasma and erythrocytes in the sensitivity analyses, which excluded one patient with surprisingly high plasma EPA concentrations.

Except for selenium, patients’ micronutrient status was good in our study. We observed higher serum folic acid and vitamin B12 concentrations in patients compared with controls, in line with Rojas-Agurto et al. [23], who studied 10 adherent adults with PKU, as well as with meta-analyses across different age groups [11,24]. Because vitamin B12 deficiency may be masked when circulating folic acid levels are elevated [25], and functional deficiency might occur even when B12 concentrations are in the normal range [26], it is important to consider more relevant biomarkers, such as total homocysteine concentrations [27]. In line with a previous report [23], total homocysteine concentrations were in the normal range of 5 to 12 µmol/L for almost all patients (32/34, 94%), thus suggesting that they were not B12 deficient in this study.

Mixed results have been reported regarding the vitamin D status between patients and controls, with meta-analyses concluding that there was no significant difference [11,24]. Like Rojas-Agurto et al. [23], we found higher vitamin D concentrations in the PKU group, with only 3/34 patients (9%) having serum levels below the recommended 50 nmol/L, against 18/34 (53%) in the control group. This is not surprising as the majority (22/34, 65%) of controls were recruited from the Netherlands in February/March, and a Dutch study reported vitamin D deficiency in close to 60% of healthy individuals in the winter [28]. Conversely, patient recruitment spread throughout the year (including spring and summer time). Furthermore, vitamin D intake was significantly higher in the PKU group, protein substitutes being the main source (~90%) of vitamin D in patients. Nevertheless, 37% of patients (11/30) did not meet the DRI for vitamin D. Worse, none of the subjects in the control group met the DRI. Others have reported normal blood vitamin D concentrations in adult patients regularly taking their protein substitutes [9,14].

We observed higher whole blood pyridoxal 5′-phosphate (PLP, the active coenzyme of vitamin B6) concentrations in the PKU group compared with controls. Published data on vitamin B6 status in PKU are scarce. We identified only two studies, in children, reporting opposite results: Prince et al. [29] found higher plasma PLP and total vitamin B6 concentrations, whereas Schulpis et al. [30] found lower plasma PLP levels, in pediatric patients compared with controls. Prince et al. suggested that the reduced protein, high bioavailability of vitamin B6 sources in the Phe-restricted diet, and fortified protein substitutes could explain the increased circulating PLP levels. Vitamin B6 intake was significantly higher in our patients (without exceeding EFSA’s tolerable upper intake levels). Conversely, 15/35 controls (44%) had intakes below the DRI.

Although significantly lower serum alpha-tocopherol (vitamin E) concentrations were observed in the PKU group compared with controls (mean difference: −6 µmol/L, or a −13% difference), concentrations were all within the reference range (15–45 µmol/L), in line with another study in adults with PKU [31]. Interestingly, 15/35 (43%) of controls had values >45 µmol/L (vs. 23% in the PKU group), suggesting they were consuming many foods naturally rich in or fortified with vitamin E.

In line with Montoya Parra et al. [11], mineral status was similar between patients and controls for calcium, ferritin, transferrin, zinc, and magnesium, whereas selenium levels were significantly lower (−16%) in the PKU group. Studies in adults with PKU regularly taking their protein substitute reported normal concentrations of iron/ferritin [9,31], as well as selenium and zinc, though levels of these two minerals were at the lower end of the normal range [9,31]. Multiple studies have described oxidative stress as a concern in patients with PKU [32]. Selenium and zinc are critical antioxidants, as is ubiquinone (also known as coenzyme Q10), for which lower concentrations were also found in this study between the PKU and control groups, consistent with previous reports [33,34]. Adequate antioxidant status in PKU is often dictated by adherence to protein substitutes, since natural food sources are limited in the Phe-restricted diet [34,35,36]. In our study, we did observe lower selenium concentrations despite adequate intake, which may be due to the bioavailability of the selenium source. One study suggested that the situation may improve with glycomacropeptide (GMP)-based protein substitutes [37], as GMP may have bioactive properties, may modulate the microbiota so that absorption might be enhanced, and/or GMP may have antioxidant properties that could spare selenium. However, further investigations are required to confirm such a hypothesis [38].

We found lower serum concentrations in the PKU group for multiple amino acids compared with the control group. Phe, along with nine other large neutral amino acids, compete for entry into the brain via the SLC7A5 transport protein (also known as the LAT1 transporter) [39]. In our study, serum concentrations of all non-Phe large neutral amino acids except one (threonine) were significantly lower in the PKU group compared with the control group. Previously, Cannet et al. [40] did not find any significant difference in plasma concentrations of branched-chain amino acids in a group of 22 treated adult patients with PKU compared with 14 healthy controls. Furthermore, glutamine concentrations were higher in the Cannet study, whereas we found no difference, and they reported similar glycine concentrations between groups, whereas we found higher concentrations in the PKU group in our study. Their method for measuring amino acid concentrations differed from our study. More recently, Matuszewska et al. [41] also found alterations in serum amino acid concentrations of adult patients with PKU, e.g., lower tyrosine and higher glycine concentrations than controls. However, there appeared to be no between-group differences in concentrations of branched-chain amino acids. Further research is needed to understand whether differences in amino acid concentrations exist and to what extent they might be clinically relevant. Other studies (without a control group) reported that blood amino acid concentrations were all in the reference range in patients who adhere to their protein substitutes [9,42].

To our knowledge, we are the first to report on the uridine status in PKU. Uridine is a constituent of nucleotides and nucleic acids, and it is a precursor of brain phosphatidylcholine in membranes. In addition to its pivotal role in the synthesis of these molecules, as a metabolite, uridine is also critically involved in circadian rhythms, inflammatory responses, antioxidant processes, and aging [43]. Lower plasma uridine levels have been reported in older adults with mild cognitive impairment, a pre-dementia stage [44]; however, the clinical relevance of the lower concentrations observed in our patients is unclear.

The lower creatinine concentrations observed in our study compared with controls are in line with Cannet et al. [40] and may be related to a lower muscle mass, although this was not measured in the present study. As creatinine is a product of creatine metabolism, lower concentrations could also be due to low meat consumption (a major source of creatine) in the Phe-restricted diet as well as reduced de novo creatine synthesis, which necessitates arginine, and arginine concentrations were lower in patients than controls. Creatinine concentrations in the normal range were reported by Hochuli et al. [9] and Prepok et al. [45].

Although two-thirds of patients appear to have magnesium intakes above EFSA’s tolerable upper intake level (250 mg/day), this level only applies to readily dissociable magnesium salts (e.g., found in over-the-counter mineral supplements), due to their low absorption rate resulting in an osmotic laxative effect [46]. Protein substitutes are fortified with magnesium salts; however, these salts are part of a complex product matrix including carbohydrates and fats. Furthermore, protein substitutes are usually taken around a meal. This will counteract the osmotic effect of magnesium salts in the gut [46], and therefore, patients’ intake levels in this study are unlikely to be a concern. As mentioned earlier, there was no clinically relevant difference in serum magnesium levels between the PKU and control groups, and all patients had values within the reference range (0.7 to 1.05 mmol/L).

Subjective cognitive wellbeing was assessed using the FACT-Cog questionnaire, a well-established questionnaire that has been translated into different languages and tested in populations other than patients with cancer, such as students and older adults [47]. Normative data published on the healthy population compared with cancer patients in France were in the same range as the present results [48]. Although the scores of the PKU group were significantly lower than the control group for three of the four subscales in our study, their scores were still similar to, or better than, the healthy controls from the French study. The most distinct difference was the subscale on ‘Comments from others’, which was not significantly different between the French healthy population and cancer patients, in contrast to our results comparing the PKU and control groups. PKU is an inherited metabolic disorder present from birth and requiring lifelong management, while cancer oftentimes presents later in life, which may explain this difference. We elected to use the FACT-Cog questionnaire because we wanted an easy-to-use cognitive assessment tool, to keep the burden on study participants to a minimum. However, we realize that this makes it challenging to compare our results with the PKU literature. In the largest meta-analysis to date in early-treated adults with PKU, Romani et al. [49] demonstrated that there are statistically significant cognitive impairments compared with controls, though the magnitude of the deficit varies across different cognitive functions. We support the recommendation of the authors to use a PKU-specific cognitive battery to assess cognition in future studies. The National PKU Alliance neurocognitive workgroup in the US has also just published a white paper on a neurocognitive assessment platform relevant to clinical trials in PKU [50].

This study has some limitations. The overall sample size was limited (N = 34 pairs), though this is common in the field of rare diseases. The PKU and control subjects were well matched for age and sex by design and, coincidentally, BMI, although not for location. While we endeavored to recruit control subjects from the same area as their counterpart with PKU, this proved too challenging in practice, and ultimately, most of the controls were recruited by an additional study site in the Netherlands. Due to the exploratory nature of this study, no corrections for multiplicity were applied in the statistical analysis. Therefore, provided *p*-values should be interpreted with caution. However, although statistical analyses are helpful, as for any other study, one also ought to consider whether any differences observed are clinically meaningful. Finally, to reduce the burden on study participants, we assessed nutrient status and intake at a single time point. Additional time points would have strengthened our conclusions, though patients with PKU are known to have fairly consistent eating habits due to the many dietary restrictions.

## 5. Conclusions

In conclusion, the nutrient status and intakes of adult patients with PKU using a protein substitute were adequate for most nutrients (DHA, micronutrients), although lower blood concentrations, compared with controls, were observed for selenium, ubiquinone, and some amino acids. The metabolic control was variable, reflecting variability in adherence with the Phe-restricted diet. The regular use of protein substitutes remains a point of attention in adults with PKU, as they are essential to meet DRIs.

## Figures and Tables

**Table 1:** Baseline characteristics.

Parameter/Statistic		PKU (N = 34)	Control (N = 35)	*p*-Value ^1^
Age (years)	Mean (SD)	28.00 (8.88)	28.54 (9.34)	0.9999
	95% CI	24.90–31.10	25.34–31.75	
	Median	25.0	25.0	
	Min–Max	18.0–54.0	18.0–57.0	
Sex	Female	16 (47.1%)	16 (45.7%)	
	Male	18 (52.9%)	19 (54.3%)	
BMI (kg/m^2^)	Mean (SD)	25.25 (6.11)	24.18 (3.85)	0.3879
	95% CI	23.12–27.39	22.85–25.50	
	Median	23.7	23.7	
	Min–Max	17.3–50.6	18.3–34.7	
Patient-specific Baseline Characteristics				
PKU phenotype	Classic PKU	33 (97.1%)		
	Moderate PKU	1 (2.9%)		
Phe tolerance (mg/day) ^2^	N	26		
	Mean (SD)	430.4 (141.4)		
	95% CI	373.3–487.5		
	Median	400.2		
	Min–Max	200.0–700.0		
Average Phe-level of 1 year ago (µmol/L)	N	34		
	Mean (SD)	668.9 (267.3)		
	95% CI	575.7–762.2		
	Median	648.9		
	Min–Max	241.0–1479.0		
Average Phe-level of 2 years ago (µmol/L)	N	34		
	Mean (SD)	631.4 (228.4)		
	95% CI	551.7–711.1		
	Median	645.5		
	Min–Max	241.8–1189.0		
Historical compliance	According to:	Metabolic team	Patient	
low-Phe diet	Never	1 (2.9%)	1 (2.9%)	
	Occasionally	3 (8.8%)	0 (0%)	
	Sometimes	6 (17.6%)	6 (17.6%)	
	Frequently	12 (35.3%)	14 (41.2%)	
	Usually	7 (20.6%)	10 (29.4%)	
	Always	5 (14.7%)	3 (8.8%)	
Current compliance low-Phe diet	Yes	22 (64.7%)		
	No	12 (35.3%)		

^1^ Results from a paired *t*-test comparing matched subjects with (N = 34) and without PKU (N = 34). ^2^ Excluding all patients from Ghent, where estimation of Phe tolerance is not completed. BMI, body mass index; CI, confidence interval; N, number; Phe, phenylalanine; PKU, phenylketonuria; SD, standard deviation.

**Table 2:** Nutrient and biochemical concentrations in PKU vs. control groups.

Statistical Difference	Parameter	Pairs (N)	Difference Mean (SD)	*p*-Value ^1^
Vitamins ^2^				
Significantly lower in PKU group	Alpha-tocopherol (Vitamin E) [µmol/L]	33	−6.03 (14.01)	0.0189
Significantly higher in PKU group	Folic Acid [nmol/L]	34	17.56 (14.12)	<0.0001
	25-OH Vitamin D Total [nmol/L]	34	31.45 (33.05)	<0.0001
	Vitamin B12 [pmol/L]	34	195.82 (259.99)	0.0001
	Pyridoxal 5′-phosphate (Vitamin B6) [nmol/L]	33	82.21 (65.45)	<0.0001
Minerals ^3^				
Significantly lower in PKU group	Selenium [µmol/L]	34	−0.18 (0.32)	0.0024
No significant difference	Calcium [mmol/L]	34	0.02 (0.09)	0.1413
	Ferritin [µg/L]	34	−10.80 (97.07)	0.5211
	Transferrin [g/L]	34	−0.12 (0.62)	0.2803
	Zinc [µmol/L]	34	1.09 (4.69)	0.1855
Significantly higher in PKU group	Magnesium [mmol/L]	34	0.02 (0.06)	0.0269
Lipids				
No significant difference	Plasma EPA (mg/L)	34	−3.80 (14.02)	0.1238
	Plasma DHA (mg/L)	34	0.49 (25.05)	0.9092
	Plasma EPA (%)	34	−0.07 (0.46)	0.3539
	Plasma DHA (%)	34	0.20 (0.77)	0.1427
	RBC EPA (%)	30	−0.12 (0.36)	0.0777
	RBC DHA (%)	30	0.22 (1.25)	0.3460
Other Parameters ^4^				
Significantly lower in PKU group	Creatinine [µmol/L]	34	−5.15 (12.51)	0.0222
	Homocysteine [µmol/L]	34	−2.85 (5.02)	0.0023
	Ubiquinone [nmol/L]	34	−312.32 (380.40)	<0.0001
	Uridine [µmol/L]	29	−0.54 (1.24)	0.0252

^1^ Results from a paired *t*-test comparing matched subjects with and without PKU. ^2^ Pyridoxal 5′-phosphate (active form of vitamin B6) was measured in whole blood; all other vitamins were measured in serum. ^3^ Selenium and zinc were measured in plasma; all other minerals were measured in serum. ^4^ Concentrations were measured in plasma with the exception of creatinine, which was measured in serum. DHA, docosahexaenoic acid; EPA, eicosapentaenoic acid; N, number; PKU, phenylketonuria; RBC, red blood cells; SD, standard deviation.

**Table 3:** Serum amino acid concentrations in PKU vs. control groups.

Statistical Difference	Parameter	Difference Mean (SD)	*p*-Value ^1^
Significantly lower in PKU group	Arginine [µmol/L]	−23.43 (23.13)	<0.0001
	Asparagine [µmol/L]	−15.28 (42.76)	0.0450
	Glutamine [µmol/L]	−80.25 (90.48)	<0.0001
	Histidine [µmol/L]	−7.84 (13.08)	0.0014
	Isoleucine [µmol/L]	−8.89 (12.82)	0.0003
	Leucine [µmol/L]	−21.21 (20.95)	<0.0001
	Lysine [µmol/L]	−24.63 (32.25)	<0.0001
	Methionine [µmol/L]	−3.30 (4.31)	<0.0001
	Proline [µmol/L]	−45.62 (129.65)	0.0482
	Tryptophan [µmol/L]	−8.36 (9.48)	<0.0001
	Tyrosine [µmol/L]	−11.42 (18.53)	0.0010
	Valine [µmol/L]	−19.08 (42.81)	0.0139
No significant difference	Alanine [µmol/L]	−6.80 (86.89)	0.6513
	Aspartic Acid [µmol/L]	−1.10 (3.92)	0.1114
	Cysteine [µmol/L]	4.50 (40.36)	0.5201
	Serine [µmol/L]	−6.30 (30.38)	0.2350
	Threonine [µmol/L]	−8.96 (42.40)	0.2263
Significantly higher in PKU group	Glycine [µmol/L]	34.92 (75.88)	0.0113
	Phenylalanine [µmol/L]	795.89 (284.60)	<0.0001

^1^ Results from a paired *t*-test comparing matched subjects with and without PKU (N = 34 pairs). PKU, phenylketonuria; SD, standard deviation.

**Table 4:** Nutrient intakes in PKU vs. control groups.

Statistical Difference	Parameter	Pairs (N)	Difference (Median)	*p*-Value ^1^	Subjects with Intake Below DRI or above UL ^2^ [N/Total N (%)]		
						PKU	Control
Vitamins							
No significant difference	Niacin (Vitamin B3) [mg/day]	29	2.9	0.0737	Below DRI	Missing ^3^	Missing ^3^
Significantly higher in PKU group	Vitamin B12 [µg/day]	28	1.8	0.0212	Below DRI	11/29 (37.9)	17/34 (50.0)
	Thiamin (Vitamin B1) [mg/day]	29	1.3	<0.0001	Below DRI	Missing ^3^	Missing ^3^
	Riboflavin (Vitamin B2) [mg/day]	29	0.8	<0.0001	Below DRI	5/30 (16.7)	20/34 (58.8)
	Pantothenic acid (Vitamin B5) [mg/day]	9	5.9	0.0039	Below DRI	1/18 (5.6)	9/12 (75.0)
	Vitamin B6 [mg/day]	29	1.6	<0.0001	Below DRI	2/30 (6.7)	15/34 (44.1)
	Vitamin C, Total Ascorbic Acid [mg/day]	29	81.3	<0.0001	Below DRI	3/30 (10.0)	19/34 (55.9)
	Vitamin D (D2 + D3) [µg/day]	29	16.2	<0.0001	Below DRI	11/30 (36.7)	34/34 (100)
	Vitamin E (alpha-tocopherol) [mg/day]	28	9.1	0.0104	Below DRI	6/28 (20.7)	14/34 (41.2)
Minerals							
No significant difference	Phosphorus [g/day]	29	0.2	0.1134	Below DRI	0/30 (0)	1/34 (2.9)
Significantly higher in PKU group	Calcium [g/day]	29	0.9	<0.0001	Below DRI	4/30 (13.3)	22/34 (64.7)
					Above UL	5/30 (16.7)	
	Copper [mg/day]	29	1.2	<0.0001	Below DRI	3/30 (10.0)	20/34 (58.8)
					Above UL	2/30 (6.7)	
	Iron [mg/day]	29	12.5	<0.0001	Below DRI	2/30 (6.7)	22/34 (64.7)
	Magnesium [mg/day]	29	179.7	0.0003	Below DRI	6/30 (20.0)	21/34 (61.8)
					Above UL ^4^	20/30 (66.7)	
	Selenium [µg/day]	29	37.0	<0.0001	Below DRI	7/30 (23.3)	30/34 (88.2)
	Zinc [mg/day]	29	9.1	<0.0001	Below DRI	4/30 (13.3)	23/34 (67.6)
					Above UL	8/30 (26.7)	
Energy and macronutrients							
Significantly lower in PKU group	20:5 *n*-3 (EPA) [mg/day]	12	−25.0	0.0039			
No significant difference	Energy [kcal/day]	29	189.7	0.3024			
	Fiber [g/day]	19	−1.8	0.4180			
	22:6 *n*-3 (DHA) [mg/day]	13	−70.0	0.0547			
	Protein [g/day]	29	−4.3	0.7439			

^1^ Results from a Wilcoxon Signed-Rank test comparing matched subjects with and without PKU. ^2^ DRI: daily recommended intake and UL: tolerable upper intake level as published by EFSA. ^3^ Dietary reference values for niacin were not used because EFSA makes a distinction between nicotinamide and nicotinic acid, and this level of detail was not available for our study. EFSA dietary reference values for thiamin are based on energy intake and were not used. ^4^ The UL for magnesium (Mg) (250 mg/day) is not applicable to Mg naturally found in foods; it only applies to readily dissociable Mg salts in food supplements. As control subjects were not taking any Mg supplements, none were above the UL. Mg in PKU protein substitutes is in the form of Mg salts but as part of a complex food matrix; see Section 4 of the manuscript for more details. DHA, docosahexaenoic acid; EPA, eicosapentaenoic acid; PKU, phenylketonuria.

**Table 5:** Intake from protein substitutes (PKU group).

Nutrient Category	Parameter	PKU Subjects N	% Intake from PS Mean (SD)
Amino Acids	Phenylalanine	30	3.46 (9.20)
	Tyrosine	18	90.30 (20.75)
Vitamins	Niacin (Vitamin B3)	30	66.30 (21.90)
	Vitamin B12	29	83.58 (14.23)
	Thiamin (Vitamin B1)	30	62.97 (20.77)
	Riboflavin (Vitamin B2)	30	67.84 (19.93)
	Pantothenic acid (Vitamin B5)	18	66.59 (23.22)
	Vitamin B6	30	62.91 (20.83)
	Vitamin C, Total Ascorbic Acid	30	42.61 (23.79)
	Vitamin D (D2 + D3)	30	89.03 (18.42)
	Vitamin E (alpha-tocopherol)	28	52.19 (22.64)
Minerals	Phosphorus	30	63.46 (17.95)
	Calcium	30	63.84 (22.23)
	Copper	30	58.47 (24.60)
	Iron	30	67.10 (19.01)
	Magnesium	30	62.02 (19.93)
	Selenium	30	70.26 (23.44)
	Zinc	30	74.29 (23.16)
Energy and Macronutrients	20:5 *n*-3 (EPA)	4	75 (50)
	Energy	30	18.58 (8.35)
	Fiber	20	7.13 (15.78)
	22:6 *n*-3 (DHA)	6	100 (0)
	Protein	30	65.57 (17.02)

DHA, docosahexaenoic acid; EPA, eicosapentaenoic acid; PKU, phenylketonuria; PS: protein substitute; SD: standard deviation.

**Table 6:** Subjective cognitive wellbeing in PKU vs. control groups.

Parameter	Statistic	PKU N = 32	Controls N = 35	*p*-Value ^1^
Perceived cognitive impairments (CogPCI)	Mean (SD)	57.75 (17.40)	64.83 (7.48)	0.0662
	95% CI	51.48–64.02	62.26–67.40	
	Median	62.5	68.0	
	Min–Max	2.0–72.0	44.0–72.0	
Perceived cognitive abilities (CogPCA)	Mean (SD)	21.31 (5.18)	24.29 (3.67)	0.0033
	95% CI	19.45–23.18	23.02–25.55	
	Median	23.0	26.0	
	Min–Max	9.0–28.0	15.0–28.0	
Comments from others (CogOth)	Mean (SD)	13.88 (4.13)	15.40 (1.46)	0.0041
	95% CI	12.39–15.36	14.90–15.90	
	Median	16.0	16.0	
	Min–Max	1.0–16.0	8.0–16.0	
Impact on quality of life (CogQOL)	Mean (SD)	13.03 (4.20)	14.80 (2.47)	0.0473
	95% CI	11.52–14.55	13.95–15.65	
	Median	15.5	16.0	
	Min–Max	0.0–16.0	6.0–16.0	

^1^* p*-value based on a Wilcoxon Signed-Rank test comparing matched subjects with and without PKU for CogPCI and CogOth, *p*-value based on paired *t*-test for CogPCA and CogQOL. N = 32 pairs. CI, confidence interval; PKU, phenylketonuria; SD, standard deviation.

## Data Availability

Data are available upon reasonable request.

## Supplementary Materials

The following supporting information can be downloaded at: https://www.mdpi.com/article/10.3390/nu16162724/s1, Methods S1: Lab Analysis of Nutrients and other Biochemicals; Figure S1: Linear Regression Analysis for Perceived Cognitive Impairment with the Phenylalanine-to-Tyrosine Ratio as Predictor, in Patients with PKU; Table S1: Dietary Reference Values according to the European Food Safety Authority.

## References

[1] Blau N., van Spronsen F.J., Levy H.L. (2010). Phenylketonuria. Lancet.

[2] Jaulent P., Charriere S., Feillet F., Douillard C., Fouilhoux A., Thobois S. (2020). Neurological manifestations in adults with phenylketonuria: New cases and review of the literature. J. Neurol..

[3] Levy H.L. (2021). Robert Guthrie and the Trials and Tribulations of Newborn Screening. Int. J. Neonatal. Screen.

[4] van Wegberg A.M.J., MacDonald A., Ahring K., Belanger-Quintana A., Blau N., Bosch A.M., Burlina A., Campistol J., Feillet F., Gizewska M. (2017). The complete European guidelines on phenylketonuria: Diagnosis and treatment. Orphanet J. Rare Dis..

[5] Vockley J., Andersson H.C., Antshel K.M., Braverman N.E., Burton B.K., Frazier D.M., Mitchell J., Smith W.E., Thompson B.H., Berry S.A. (2014). Phenylalanine hydroxylase deficiency: Diagnosis and management guideline. Genet. Med..

[6] Dixon M., MacDonald A., White F.J. (2020). Disorders of Amino Acid Metabolism, Organic Acidaemias and Urea Cycle Disorders. Clinical Paediatric Dietetics.

[7] Brown C.S., Lichter-Konecki U. (2016). Phenylketonuria (PKU): A problem solved?. Mol. Genet. Metab. Rep..

[8] Jurecki E.R., Cederbaum S., Kopesky J., Perry K., Rohr F., Sanchez-Valle A., Viau K.S., Sheinin M.Y., Cohen-Pfeffer J.L. (2017). Adherence to clinic recommendations among patients with phenylketonuria in the United States. Mol. Genet. Metab..

[9] Hochuli M., Bollhalder S., Thierer C., Refardt J., Gerber P., Baumgartner M.R. (2017). Effects of Inadequate Amino Acid Mixture Intake on Nutrient Supply of Adult Patients with Phenylketonuria. Ann. Nutr. Metab..

[10] Rohde C., von Teeffelen-Heithoff A., Thiele A.G., Arelin M., Mutze U., Kiener C., Gerloff J., Baerwald C., Schultz S., Heller C. (2014). PKU patients on a relaxed diet may be at risk for micronutrient deficiencies. Eur. J. Clin. Nutr..

[11] Montoya Parra G.A., Singh R.H., Cetinyurek-Yavuz A., Kuhn M., MacDonald A. (2018). Status of nutrients important in brain function in phenylketonuria: A systematic review and meta-analysis. Orphanet J. Rare Dis..

[12] MacDonald A., van Wegberg A.M.J., Ahring K., Beblo S., Belanger-Quintana A., Burlina A., Campistol J., Coskun T., Feillet F., Gizewska M. (2020). PKU dietary handbook to accompany PKU guidelines. Orphanet J. Rare Dis..

[13] Wagner L.I., Sweet J., Butt Z., Lai J.-s., Cella D.J.J.S.O. (2009). Measuring patient self-reported cognitive function: Development of the functional assessment of cancer therapy-cognitive function instrument. J. Support. Oncol..

[14] Lage S., Bueno M., Andrade F., Prieto J.A., Delgado C., Legarda M., Sanjurjo P., Aldamiz-Echevarria L.J. (2010). Fatty acid profile in patients with phenylketonuria and its relationship with bone mineral density. J. Inherit. Metab. Dis..

[15] EFSA Panel on Dietetic Products, Nutrition and Allergies (NDA) (2010). Scientific Opinion on the substantiation of health claims related to docosahexaenoic acid (DHA) and maintenance of normal (fasting) blood concentrations of triglycerides (ID 533, 691, 3150), protection of blood lipids from oxidative damage (ID 630), contribution to the maintenance or achievement of a normal body weight (ID 629), brain, eye and nerve development (ID 627, 689, 704, 742, 3148, 3151), maintenance of normal brain function (ID 565, 626, 631, 689, 690, 704, 742, 3148, 3151), maintenance of normal vision (ID 627, 632, 743, 3149) and maintenance of normal spermatozoa motility (ID 628) pursuant to Article 13(1) of Regulation (EC) No 1924/2006. EFSA J..

[16] EFSA Panel on Dietetic Products, Nutrition and Allergies (NDA) (2010). Scientific Opinion on the substantiation of health claims related to eicosapentaenoic acid (EPA), docosahexaenoic acid (DHA), docosapentaenoic acid (DPA) and maintenance of normal cardiac function (ID 504, 506, 516, 527, 538, 703, 1128, 1317, 1324, 1325), maintenance of normal blood glucose concentrations (ID 566), maintenance of normal blood pressure (ID 506, 516, 703, 1317, 1324), maintenance of normal blood HDL-cholesterol concentrations (ID 506), maintenance of normal (fasting) blood concentrations of triglycerides (ID 506, 527, 538, 1317, 1324, 1325), maintenance of normal blood LDL-cholesterol concentrations (ID 527, 538, 1317, 1325, 4689), protection of the skin from photo-oxidative (UV-induced) damage (ID 530), improved absorption of EPA and DHA (ID 522, 523), contribution to the normal function of the immune system by decreasing the levels of eicosanoids, arachidonic acid-derived mediators and pro-inflammatory cytokines (ID 520, 2914), and “immunomodulating agent” (4690) pursuant to Article 13(1) of Regulation (EC) No 1924/2006. EFSA J..

[17] Calder P.C. (2012). Mechanisms of action of (n-3) fatty acids. J. Nutr..

[18] Koletzko B., Beblo S., Demmelmair H., Hanebutt F.L. (2009). Omega-3 LC-PUFA supply and neurological outcomes in children with phenylketonuria (PKU). J. Pediatr. Gastroenterol. Nutr..

[19] Plourde M., Cunnane S.C. (2007). Extremely limited synthesis of long chain polyunsaturates in adults: Implications for their dietary essentiality and use as supplements. Appl. Physiol. Nutr. Metab..

[20] Infante J.P., Huszagh V.A. (2001). Impaired arachidonic (20:4n-6) and docosahexaenoic (22:6n-3) acid synthesis by phenylalanine metabolites as etiological factors in the neuropathology of phenylketonuria. Mol. Genet. Metab..

[21] Lohner S., Fekete K., Decsi T. (2013). Lower n-3 long-chain polyunsaturated fatty acid values in patients with phenylketonuria: A systematic review and meta-analysis. Nutr. Res..

[22] Htun P., Nee J., Ploeckinger U., Eder K., Geisler T., Gawaz M., Bocksch W., Fateh-Moghadam S. (2015). Fish-Free Diet in Patients with Phenylketonuria Is Not Associated with Early Atherosclerotic Changes and Enhanced Platelet Activation. PLoS ONE.

[23] Rojas-Agurto E., Leal-Witt M.J., Arias C., Cabello J.F., Bunout D., Cornejo V. (2023). Muscle and Bone Health in Young Chilean Adults with Phenylketonuria and Different Degrees of Compliance with the Phenylalanine Restricted Diet. Nutrients.

[24] Bokayeva K., Jamka M., Walkowiak D., Dus-Zuchowska M., Herzig K.H., Walkowiak J. (2024). Vitamin Status in Patients with Phenylketonuria: A Systematic Review and Meta-Analysis. Int. J. Mol. Sci..

[25] Malouf M., Grimley E.J., Areosa S.A. (2003). Folic acid with or without vitamin B12 for cognition and dementia. Cochrane Database Syst. Rev..

[26] Walter J.H. (2011). Vitamin B12 deficiency and phenylketonuria. Mol. Genet. Metab..

[27] Vugteveen I., Hoeksma M., Monsen A.L., Fokkema M.R., Reijngoud D.J., van Rijn M., van Spronsen F.J. (2011). Serum vitamin B12 concentrations within reference values do not exclude functional vitamin B12 deficiency in PKU patients of various ages. Mol. Genet. Metab..

[28] Boonman-de Winter L.J., Albersen A., Mohrmann K., Bakx-van Baal C.M., Meijer Timmerman Thijssen D.W., Bressers J.P. (2015). High prevalence of vitamin D deficiency in the south-west Netherlands. Ned. Tijdschr. Geneeskd..

[29] Prince A.P., Leklem J.E. (1994). Vitamin B-6 status of school-aged patients with phenylketonuria. Am. J. Clin. Nutr..

[30] Schulpis K.H., Karikas G.A., Papakonstantinou E. (2002). Homocysteine and other vascular risk factors in patients with phenylketonuria on a diet. Acta Paediatr..

[31] Das A.M., Goedecke K., Meyer U., Kanzelmeyer N., Koch S., Illsinger S., Lucke T., Hartmann H., Lange K., Lanfermann H. (2014). Dietary habits and metabolic control in adolescents and young adults with phenylketonuria: Self-imposed protein restriction may be harmful. JIMD Rep..

[32] Rocha J.C., Martins M.J. (2012). Oxidative stress in phenylketonuria: Future directions. J. Inherit. Metab. Dis..

[33] Montero R., Yubero D., Salgado M.C., González M.J., Campistol J., O’Callaghan M.D.M., Pineda M., Delgadillo V., Maynou J., Fernandez G. (2019). Plasma coenzyme Q10 status is impaired in selected genetic conditions. Sci. Rep..

[34] Artuch R., Colome C., Sierra C., Brandi N., Lambruschini N., Campistol J., Ugarte D., Vilaseca M.A. (2004). A longitudinal study of antioxidant status in phenylketonuric patients. Clin. Biochem..

[35] Colome C., Artuch R., Vilaseca M.A., Sierra C., Brandi N., Lambruschini N., Cambra F.J., Campistol J. (2003). Lipophilic antioxidants in patients with phenylketonuria. Am. J. Clin. Nutr..

[36] Gassio R., Artuch R., Vilaseca M.A., Fuste E., Colome R., Campistol J. (2008). Cognitive functions and the antioxidant system in phenylketonuric patients. Neuropsychology.

[37] Daly A., Evans S., Chahal S., Santra S., Pinto A., Jackson R., Gingell C., Rocha J., Van Spronsen F.J., MacDonald A. (2019). Glycomacropeptide: Long-term use and impact on blood phenylalanine, growth and nutritional status in children with PKU. Orphanet J. Rare Dis..

[38] Allergies N, EFSA Panel on Dietetic Products (2014). Scientific Opinion on Dietary Reference Values for selenium. efsa J..

[39] Smith Q.R. (1991). The blood-brain barrier and the regulation of amino acid uptake and availability to brain. Adv. Exp. Med. Biol..

[40] Cannet C., Pilotto A., Rocha J.C., Schafer H., Spraul M., Berg D., Nawroth P., Kasperk C., Gramer G., Haas D. (2020). Lower plasma cholesterol, LDL-cholesterol and LDL-lipoprotein subclasses in adult phenylketonuria (PKU) patients compared to healthy controls: Results of NMR metabolomics investigation. Orphanet J. Rare Dis..

[41] Matuszewska E., Matysiak J., Kaluzny L., Walkowiak D., Plewa S., Dus-Zuchowska M., Rzetecka N., Jamka M., Klupczynska-Gabryszak A., Piorunek M. (2024). Amino Acid Profile Alterations in Phenylketonuria: Implications for Clinical Practice. Metabolites.

[42] Green B., Browne R., Firman S., Hill M., Rahman Y., Hansen K.K., Adam S., Skeath R., Hallam P., Herlihy I. (2019). Nutritional and metabolic characteristics of UK adult phenylketonuria patients with varying dietary adherence. Nutrients.

[43] Yang Y., Ye Y., Deng Y., Gao L. (2024). Uridine and its role in metabolic diseases, tumors, and neurodegenerative diseases. Front. Physiol..

[44] van Wijk N., Slot R.E.R., Duits F.H., Strik M., Biesheuvel E., Sijben J.W.C., Blankenstein M.A., Bierau J., van der Flier W.M., Scheltens P. (2017). Nutrients required for phospholipid synthesis are lower in blood and cerebrospinal fluid in mild cognitive impairment and Alzheimer’s disease dementia. Alzheimers Dement..

[45] Prepok F.F., Schnabel K.K., Sumanszki C., Barta A.G., Tisler A., Reismann P. (2024). Long-Term Renal Function in Adult Patients with Phenylketonuria. Nephron.

[46] Haugen M., Lillegaard I.T.L., Strand T.A., Frøyland L., Holvik K., Løvik M., Tell G.S., Iversen P.O.J.V.R. (2016). CKM Report: Risk Assessment of Magnesium in Food Supplements; Opinion of the Panel on Nutrition, Dietetic Products, Novel Food and Allergy of the Norwegian Scientific Committee for Food Safety. https://vkm.no/download/18.773639b215c8657f2a497477/1498138111265/f9ef23feb7.pdf.

[47] Costa D.S.J., Loh V., Birney D.P., Dhillon H.M., Fardell J.E., Gessler D., Vardy J.L. (2018). The Structure of the FACT-Cog v3 in Cancer Patients, Students, and Older Adults. J. Pain Symptom. Manag..

[48] Lange M., Heutte N., Morel N., Eustache F., Joly F., Giffard B. (2016). Cognitive complaints in cancer: The French version of the Functional Assessment of Cancer Therapy-Cognitive Function (FACT-Cog), normative data from a healthy population. Neuropsychol. Rehabil..

[49] Romani C., Olson A., Aitkenhead L., Baker L., Patel D., Spronsen F.V., MacDonald A., Wegberg A.V., Huijbregts S. (2022). Meta-analyses of cognitive functions in early-treated adults with phenylketonuria. Neurosci. Biobehav. Rev..

[50] Waisbren S.E., Christ S.E., Bilder D.A., Bjoraker K.J., Bolton S., Chamberlin S., Grant M.L., Janzen D.M., Katz R., Lubliner E. (2024). Neurocognitive assessment platform for clinical trials in PKU: White paper developed by the NPKUA neurocognitive workgroup. Mol. Genet. Metab..

