# Supplementary material for: Nutrient Status and Intakes of Adults with Phenylketonuria
Source: Nutrients. 2024 Aug 15;16(16):2724. doi: 10.3390/nu16162724 (PMC11357144; doi:10.3390/nu16162724)
Supplement: Supplementary file 1 [file nutrients-16-02724-s001.zip › Figure S1.pdf]

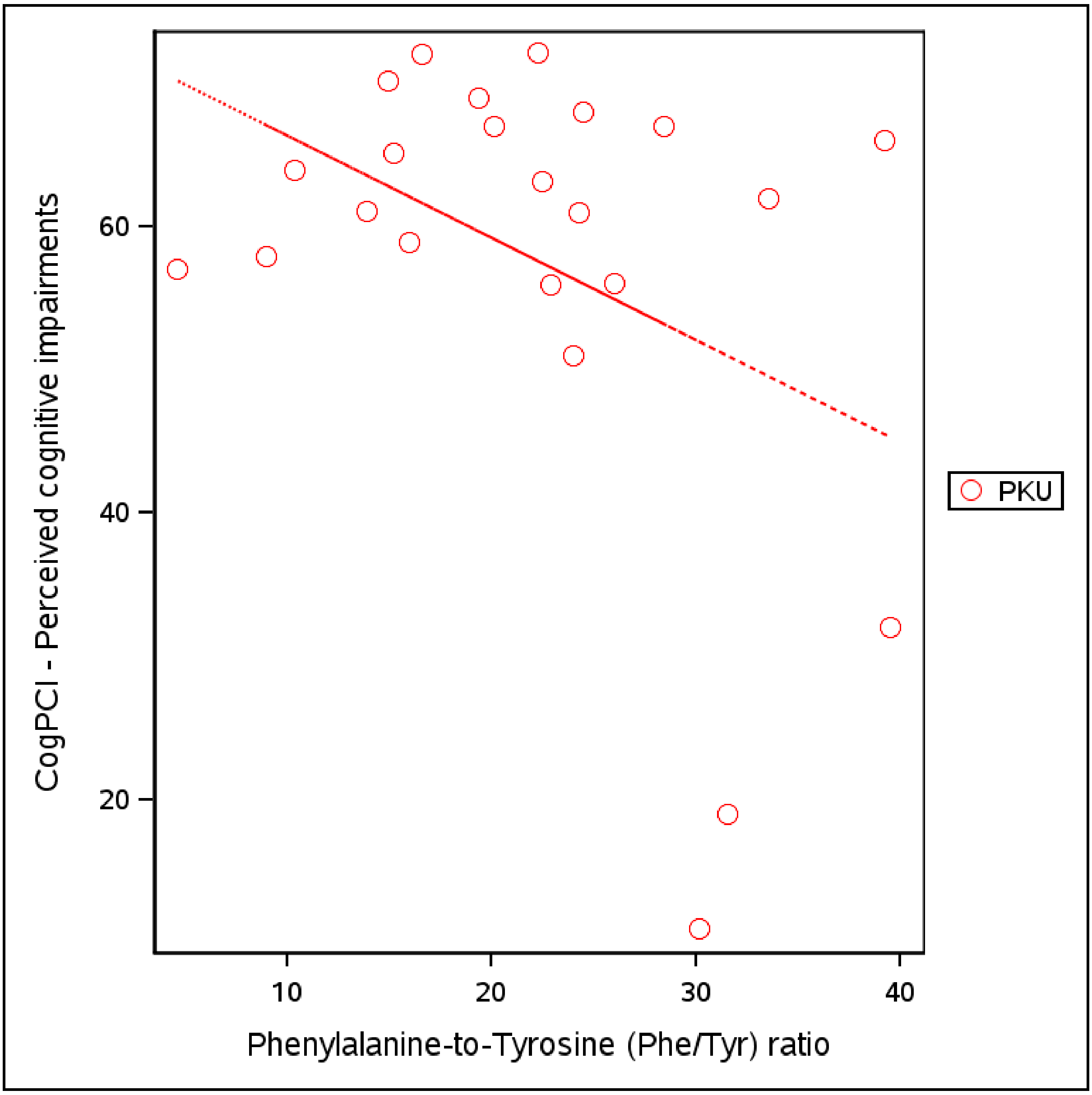


**Figure S1.** Linear Regression Analysis for Perceived Cognitive Impairment with the Phenylalanine-to-Tyrosine Ratio as Predictor, in Patients with PKU.
