# Supplementary material for: Nutrient Status and Intakes of Adults with Phenylketonuria
Source: Nutrients. 2024 Aug 15;16(16):2724. doi: 10.3390/nu16162724 (PMC11357144; doi:10.3390/nu16162724)
Supplement: Supplementary file 1 [file nutrients-16-02724-s001.zip › Methods S1.pdf]

Methods S1. Lab Analysis of Nutrients and other Biochemicals

Venous blood was collected from subjects in a fasting state during the study visit. Blood samples were collected in five types of containers: 10 mL serum, 6 mL with lithium heparin, 4 mL with K2EDTA, 6 mL for trace metal determination, and 2 mL for homocysteine detection. All nutrients and other biochemical parameters measured are listed below, including in which matrix the parameters were measured and what analysis method was used. Part of the parameters were measured by the analytical lab of Danone Research & Innovation, and part by the Reinier Haga Medical Diagnostic Centre (RHMDC).

Blood Nutrients and Other Biochemical Parameters

**Parameter**	**Matrix (Container)**[a]	**Method**	**Output (Unit)**	**Laboratory**
Uridine	Serum (1)	HPLC/UV	µmol/L	Danone
Homocysteine	Plasma (5)	HPLC/Flu	µmol/L	Danone
DHA and EPA in RBC	RBC (3)	GC/FID	FA as % of total	Danone
DHA and EPA in plasma	Plasma (3)	GC/FID	FA as % of total	Danone
Alpha-tocopherol	Serum (1)	HPLC/Flu	µmol/L	Danone
Amino acid profile[b]	Serum (1)	HPLC/Flu	µmol/L	Danone
Proline	Serum (1)	HPLC/Flu	µmol/L	Danone
Cysteine	Serum (1)	HPLC/Flu	µmol/L	Danone
Calcium	Serum (1)	Colorimetric	mmol/L	RHMDC
Folic acid	Serum (1)	CPBL	nmol/L	RHMDC
Vitamin B12	Serum (1)	CPBL	pmol/L	RHMDC
25-OH Vitamin D	Serum (1)	ILMA	nmol/L	RHMDC
Magnesium	Serum (1)	Colorimetric	mmol/L	RHMDC
Creatinine	Serum (1)	Colorimetric	µmol/L	RHMDC
Transferrin	Serum (1)	Turbimetric	g/L	RHMDC
Ferritin	Serum (1)	ILMA	µg/L	RHMDC
Ubiquinone	Plasma (2)	HPLC/Flu	nmol/L	RHMDC
Vitamin B6	Whole blood (3)	HPLC/Flu	nmol/L	RHMDC
Selenium	Plasma (4)	AAS	µmol/L	RHMDC
Zinc	Plasma (4)	AAS	µmol/L	RHMDC

[a] Containers: (1) serum, (2) with lithium heparin, (3) with K2EDTA, (4) for trace metal determination, (5) for homocysteine detection

[b] Including amino acids: asp, asn, ser, gln, his, gly, thr, arg, ala, tyr, val, met, trp, phe, ile, leu, lys

AAS = atomic absorption spectroscopy; CPBL = competitive protein binding ligand; DHA = docosahexaenoic acid; EPA = eicosapentaenoic acid; FA = fatty acid; FID = flame ionization detector; Flu = fluorescence detector; GC = gas chromatography; HPLC = high performance liquid chromatography; ILMA = immunoluminometric assay; RBC = red blood cell; RHMDC = Reinier Haga Medical Diagnostic Centre; UV = ultraviolet
