# Supplementary material for: Nutrient Status and Intakes of Adults with Phenylketonuria
Source: Nutrients. 2024 Aug 15;16(16):2724. doi: 10.3390/nu16162724 (PMC11357144; doi:10.3390/nu16162724)
Supplement: Supplementary file 1 [file nutrients-16-02724-s001.zip › Table S1.pdf]

**Table S1. Dietary Reference Values according to the European Food Safety Authority**

| Category | Nutrient parameter | Parameter | Age | Sex | AI | PRI | UL |
|---|---|---|---|---|---|---|---|
| Minerals | Calcium | Ca result (g/day) | 18-24 years | M/F | NA | 1000 mg/day | 2500 mg/day |
| Minerals | Calcium | Ca result (g/day) | ≥ 25 years | M/F | NA | 950 mg/day | 2500 mg/day |
| Minerals | Copper | Copper result (mg/day) | ≥ 18 years | M | 1.6 mg/day | NA | 5 mg/day |
| Minerals | Copper | Copper result (mg/day) | ≥ 18 years | F | 1.3 mg/day | NA | 5 mg/day |
| Minerals | Iron | Ferritin result (mg/day) | ≥ 18 years | F | NA | 16 mg/day | ND |
| Minerals | Iron | Ferritin result (mg/day) | ≥ 40 years | F | NA | 11 mg/day | ND |
| Minerals | Iron | Ferritin result (mg/day) | ≥ 18 years | M | NA | 11 mg/day | ND |
| Minerals | Magnesium | Magnesium (mg/day) | ≥ 18 years | M | 350 mg/day | NA | 250 mg/day |
| Minerals | Magnesium | Magnesium (mg/day) | ≥ 18 years | F | 300 mg/day | NA | 250 mg/day |
| Minerals | Phosphorus | Phosphorus (g/day) | ≥ 18 years | M/F | 550 mg/day | NA | ND |
| Minerals | Selenium | Selenium result (µg/day) | ≥ 18 years | M/F | 70 µg/day | NA | 300 µg/day |
| Minerals | Zinc | Zinc result (mg/day) | ≥ 18 years | M | NA | 11.7 mg/day | 25 mg/day |
| Minerals | Zinc | Zinc result (mg/day) | ≥ 18 years | F | NA | 9.3 mg/day | 25 mg/day |
| Vitamins | Vitamin B12 | Vitamin B12 result (µg/day) | ≥ 18 years | M/F | 4 µg/day | NA | ND |
| Vitamins | Pantothenic acid | Pantothenic acid (Vitamin B5) (mg/day) | ≥ 18 years | M/F | 5 mg/day | NA | ND |
| Vitamins | Riboflavin | Riboflavin (Vitamin B2) (mg/day) | ≥ 18 years | M/F | NA | 1.6 mg/day | ND |
| Vitamins | Vitamin B6 | Vitamin (B6) result (mg/day) | ≥ 18 years | M | NA | 1.7 mg/day | 25 mg/day |
| Vitamins | Vitamin B6 | Vitamin (B6) result (mg/day) | ≥ 18 years | F | NA | 1.6 mg/day | 25 mg/day |
| Vitamins | Vitamin C | Vitamin C, total ascorbic acid (mg/day) | ≥ 18 years | M | NA | 110 mg/day | ND |
| Vitamins | Vitamin C | Vitamin C, total ascorbic acid (mg/day) | ≥ 18 years | F | NA | 95 mg/day | ND |
| Vitamins | Vitamin D | Vitamin D (D2 + D3) (µg/day) | ≥ 18 years | M | 15 µg/day | NA | 100 µg/day |
| Vitamins | Vitamin D | Vitamin D (D2 + D3) (µg/day) | ≥ 18 years | F | 15 µg/day | NA | 100 µg/day |

Note: either the AI or PRI was used as daily recommended intakes, whichever was available.

AI = adequate intakes; F = female; M = male; NA = not available; ND = not defined; PRI = population reference intakes; UL = tolerable upper intake level
